# Estimating the direct medical cost of illness of COVID-19 hospitalisations in Kuwait: efficiency trade-offs from real-world data analysis

**DOI:** 10.1186/s13561-025-00694-9

**Published:** 2025-11-19

**Authors:** Mohammad Almari, Anna Vassall, Stephen O’Neill, Zia Sadique

**Affiliations:** 1 Department of Health Policy & Management, College of Public Health, Kuwait University, Shadadiyah, Kuwait; 2 Department of Health Services Research & Policy, London School of Hygiene and Tropical Medicine, University of London, London, UK; 3 Economic Evaluation and analysis Unit, World Health Organisation, Geneva, Switzerland

**Keywords:** Cost of illness, COVID-19, Hospitalization, Economics, Efficiency, Real-world data, Kuwait

## Abstract

**Background:**

COVID – 19 has had a profound impact on the economy, health systems within countries, and individuals around the world. To provide insight that may enhance the preparedness for future pandemics, a comprehensive cost assessment is vital. This study aims to estimate the direct cost of illness (CoI), as well as the national burden of treating hospitalised COVID-19 patients.

**Methods:**

This study is prevalence-based retrospective study containing all patients admitted to a single designated hospital in Kuwait for the treatment of COVID-19. Micro (bottom-up) and macro (top-down) costing methods were used to evaluate direct medical CoI from a hospital perspective. Cost components were grouped as consumables, equipment, and human resources, and sensitivity analysis was used to account for uncertainty of inputs. The cost per admission was reported in local currency and international dollars (PPP$).

**Results:**

Data on 7569 patients was analysed, 52.8% of whom were male, 69.2% were above 41 years, 22% had previously vaccinated for COVID-19, 22% were admitted to the ICU, and 18% had ≥ 3 pre-existing comorbidities. The mean CoI per admission was 12,063 PPP$, with overheads accounting for 45% of this figure, while consumables, human resources, and equipment accounted for 30%, 19%, and 7%, respectively. The sensitivity analysis demonstrated that overall cost uncertainty was primarily driven by variations in human resource costs rather than by uncertainties related to personal protective equipment (PPE) or ventilator use.

**Conclusion:**

The substantial economic impact of COVID-19 on Kuwait’s healthcare system has emphasised the significant role human resource costs has on overall expenditure. These findings provide valuable insights for future pandemic preparedness.

**Supplementary Information:**

The online version contains supplementary material available at 10.1186/s13561-025-00694-9.

## Background

The COVID-19 pandemic, caused by the SARS-CoV-2 virus, imposed an unprecedented economic burden on healthcare systems worldwide. Up to March 2025, the COVID-19 pandemic had resulted in over 700 million cumulative cases, in addition to 6.9 million mortalities globally straining both health systems and economies alike since its designation as a pandemic by the World Health Organisation on March 11, 2020 [1–3]. Beyond the immediate health crisis, the pandemic exposed vulnerabilities in healthcare financing and resource allocation, with direct medical costs such as those for hospitalisation, which have emerged as a critical challenge for policymakers [4–6]. Understanding these costs is essential not only for managing the ongoing effect of the crisis, but also to aid in building resilient healthcare systems capable of withstanding future pandemics.

Due to its magnitude and variability, the direct costs associated with managing COVID-19 patients, in particular hospitalisation costs, have been a significant concern for policymakers and healthcare providers [7]. Understanding direct cost is not only essential during the time of crisis but also in the post crisis phase, as it significantly contributes to enhancing preparedness and resilience. Not only does it reflect treatment expenses but the broader strain any crisis has on healthcare infrastructure, including staffing, equipment, and consumables. Despite its significance, early in the pandemic, the urgency of the response from the Kuwait public health system limited the ability to conduct comprehensive cost analyses, leaving gaps in understanding the true economic burden [8]. This lack of data hindered effective budgeting and resource planning, underscoring the need for detailed cost insights to initiate both immediate responses and long-term preparedness strategies. An important aspect of the Kuwaiti response plan was the allocation of a single hospital for all COVID-19 cases as planned by Kuwait Ministry of Health (MOH) officials. Kuwait operates a predominantly publicly funded healthcare system, with the Ministry of Health as the main provider and financier of services. Public hospitals deliver carefree of charge to nationals and at subsidised rates to residents, ensuring broad access but placing substantial fiscal pressure on the government during large-scale health crises. Understanding the costs associated with this approach can inform long-term preparedness strategies and improve financial planning for future pandemics.

To inform long-term preparedness, a thorough understanding of the direct costs of treating COVID-19 in Kuwait is imperative. Initial studies were scarce due to the pandemic’s emergency context, but subsequent research has revealed significant variations by healthcare system structures, unit costs, clinical pathways and disease severity [5, 7, 9–12]. For instance, two studies from Iran reported mean direct costs ranging from $426 to $3,755 [13, 14], while in China, costs escalated with disease severity, peaking for critically ill patients to $52,432 [15]. In the United States, the costs per admission reached $12,046 [16], and had increased in another study by Bartch et al. to $14,366 [17]. A study in South Africa observed that cost and resource use increased as disease severity evolved [18, 19]. This was supported by other findings that relate higher resource use with the most critically ill patients [20–25].

Regionally, a study in Saudi Arabia showed that the mean cost per patient ranged from $11,387 to $21,178 for the general ward and ICU respectively [26]. In contrast, our previous study in Kuwait estimated the mean cost of hospitalisation was $7,344 for the management of COVID-19 inpatients [27]. Although this was the first attempt at estimating the direct cost in a local setting, our assessment was significantly limited by several factors: [1] the small patient cohort (*n* = 485) reduced statistical power and prevented analysis of cost variations across different patient subgroups and disease severities; [2] the restricted three-month study period captured only a single pandemic wave, failing to account for temporal variations in resource utilisation and costs across different phases of the pandemic; and [3] the step-down costing approach used provided only aggregated cost estimates without the granular patient-level resource consumption data needed for detailed economic analysis. Another recent study in Kuwait has estimated the mean cost of hospital stays at $16,373 per hospitalised patient [28]. This study estimated the Cost of Illness (CoI) over three months during the first wave utilising hospital charges rather than economic costs, which fails to capture the true opportunity costs of resources diverted to COVID-19 care and potentially misrepresents the actual economic burden on the healthcare system. These methodological limitations in existing studies highlight the need for a more comprehensive economic cost analysis using patient-level data over a longer timeframe. Understanding these cost differences is crucial for resource allocation, international cooperation and pandemic preparedness.

The abovementioned cost estimates across different healthcare systems highlights the heterogeneity of COVID-19 hospitalisation costs both across and within countries [7, 29]. Resource quantification methods varied from using parametric modelling of resource inputs [17] to using different costing approaches such as bottom-up approach [15, 18, 30–32], top-down [33], or both [27, 34]. Current knowledge gaps in evaluating COVID-19 hospitalisation costs undermine long-term pandemic preparedness since efficient decision making requires the weighing up of costs and benefits. While financial costs (e.g., direct expenditure on healthcare services) are vital for immediate budgeting, economic costs – including the opportunity costs of diverted resources – provide a fuller picture for strategic planning [35, 36]. There is a knowledge gap in estimating the economic costs of COVID-19 hospitalisation in both regional and local settings. While some regional studies have estimated hospitalisation costs, they have particularly focused on financial costs – such as hospital charges, while neglecting broader economic costs, including opportunity costs tied to resource utilisation [13, 26–28]. Such studies prioritise financial metrics over comprehensive economic analyses, potentially underestimating the true burden of the disease [37]. Moreover, the granular resource use that comprises hospitalisation costs were unclear in the previous local studies [7, 8].

To this end, this study addresses such deficiencies by focusing on individualised, region-specific cost data in Kuwait, aiming to quantify the economic costs of COVID-19 hospitalisation in 2021. By doing so, it seeks to bridge the gap between short-term financial planning and long-term economic preparedness, offering insights applicable to Kuwait and beyond its region. To our knowledge, this is the first study in Kuwait to use comprehensive patient-level data over an entire year to estimate the economic, rather than solely financial, cost of COVID-19 hospitalisation, providing a more accurate basis for policy and preparedness planning. This study aims to estimate the cost of COVID-19 hospitalisations using retrospective CoI study and state-of-the-art costing guidelines [35]. The secondary aim is to estimate the overall national health economic burden of COVID-19 for all hospitalised patients in Kuwait during 2021.

## Methods

### Study design, setting, and participants

This retrospective observational CoI study utilises a combination of micro-costing (bottom-up) and macro-costing (top-down) approaches. Specifically, the study was conducted in Jaber Al-Ahmed Hospital, a newly established hospital in Kuwait, with a bed capacity of 1168, that emerged as a task – specific hospital treating COVID-19 patients exclusively. A single-centre design was selected because this hospital served as the sole national referral facility for all COVID-19 admissions during the study period, ensuring complete case capture; however, this may limit generalisability to other healthcare settings. Patient-level data such as demographics, clinical, diagnostic, and therapeutic data was extracted from electronic health records (EHRs) available within the hospital health information system, to estimate the direct cost of treating COVID-19 cases per admission. Costs were calculated from the provider perspective, and the cost estimation aligned with the Global Health Costing Consortium Reference Case for Estimating the Cost of Global Health Services and Interventions guidelines ^(35)^. The analysis was restricted to direct medical costs to allow for precise estimation of the hospital-based economic burden using detailed patient-level resource use data. Mortality-related and other indirect costs were excluded because their estimation requires different data sources, such as national mortality databases, labour market statistics, and long-term outcome projections, which were beyond the scope and data availability of this study. The study was approved by the Research and Ethics Committee of the Kuwait MOH and the Standing Committee for Coordination of Health and Medical Research (Approval Code:1502/2020). The requirement for informed patient consent was waived in accordance with Kuwait MOH regulations and institutional policies, as the study involved secondary analysis of de-identified routinely collected data.

All patients admitted between January 1 st and December 31 st, 2021, were included in the study. No sampling was performed, as the study population comprised the complete census of all eligible COVID-19 admissions to the designated hospital during the study period. The eligibility criteria for admission to the hospital was a confirmed, positive polymerase chain reaction (PCR). The inclusion criteria for the study were all eligible admissions during the study period. with an available admission/discharge date and a definitive discharge status (discharged alive/died). A total of 7569 patients were included in the analysis.

### Data sources, measurements and resources valuation

A combination of three sources of information were utilised to identify, measure and value of the services provided; [1] EHRs [2], MOH Budget Control and Financial Affairs sector, and [3] General Medical Stores Department for procurement and tenders for the following items: medications, therapeutic agents, medical consumables and medical equipment/devices. The approach followed in the acquisition of data from the above-mentioned sources was [1] measuring the actual resources used by reviewing EHRs [2], evaluating the COVID-19 Adult Management Protocol as a reliable source of clinical management care when further information is needed, and [3] through expert opinion to complement any ambiguity in previous sources. We obtained the costing data of consumables (non-durables with a shelf-life of less than one year), capital equipment with a shelf-life of more than one year (equipment, building, medical devices), and human resources costs. Certain clinical services, such as respiratory therapy, were not consistently recorded in the EHR and were therefore absent from the primary dataset; their potential impact on costs was explored in sensitivity analyses by including ventilator-related costs.

The resource use is grouped into three broad categories (consumables, human resources, and capital equipment, and overheads). The mean cost per admission was calculated as the sum of consumables, human resources, equipment and overheads costs for each patient divided by all admissions. We reported economic costs rather than the financial costs in our cost estimates to capture the full value of resources used to treat COVID-19 patients. Further description about the inputs used in estimating the cost per patient for each resource category by each level of care – general wards and ICUs, are included in the additional files. Each item of resource use was valued using the corresponding unit costs and multiplied by the quantity of resources used. Costs are presented in Kuwaiti Dinars (KD) and converted to international Dollars using the Purchasing Power Parity ($PPP) calculated for June 2021 (1 KD = 5.2 $PPP) for international comparisons [38].

### Consumables

Consumables are non-durable items that cannot be reused and are recurrent. The resource inputs and their respective unit costs were categorised into the following categories: diagnostic tests, medications, and personal protective equipment (PPEs). Apart from PPEs, the consumables outlined in [Additional file 1] reflect the actual resource use of services for each patient from their medical records. Thus, a sensitivity analysis was used to vary the use of PPEs with (± 50%) from the base case assumption as described in [Additional file 1] (Table S3). The unit costs presented in the file were obtained from Kuwait’s MOH procurement documents.

### Capital equipment

In our study, capital equipment is defined as any resource that has a useful life and can be utilised for more than one year including medical devices, beds, and ventilators, and associated costs are calculated as the cost per day. The resources used during hospitalistion were identified, and the quantities of resources were grouped according to level of care (general ward/ICU). All equipment was set to have a useful life of ten years based on the biomedical engineering department as per MOH guidelines. Capital items with a useful life exceeding 10 years were accounted for within the overhead cost category to avoid double-counting in the capital equipment calculations. Thus, all equipment costs were annualised by applying the annualisation factor corresponding to ten years of useful life. The unit cost of the item was divided by the annualisation factor (7.72%) with a discount rate of 5% to estimate the cost per year [39]. The resources, quantity, and unit costs along with the calculations per patient day is further detailed in [Additional file 2 see Tables S4 & S5]. This approach provides economic cost of inputs and depicts the opportunity cost forgone by allocating hospital services solely to treating COVID-19, following the guidelines in Creese et al. [36].

### Human resources

The inputs for the human resource costs were derived from the time spent for each cadre involved in medical care as detailed in [Additional file 3]. The annual salaries were obtained from the above sources, and the breakdown of daily and hourly wages were calculated. The total working hours per week were assumed to be 40 h, and the cost per hour for both remuneration schemes (national/non-national staff) was presented. The nationals/non-nationals ratio (1/3), which is based on the MOH health workforce data, was used to estimate the weighted cost per hour in the base case scenario since costs differ by nationality as wage rates are typically higher among Kuwaiti nationals [Additional file 3 see Table S6]. In the sensitivity analysis, the upper and lower bounds of the highest and lowest wage schemes for both groups were used, calculated on an hourly and minute basis. These bounds were determined by referring to the remuneration scheme provided by the MOH finance department, allowing for the calculation of the wage per minute for the base case as well as the highest and lowest limits.

### Overheads

The cost of overheads, which is the cost of preparing a setting where health services can be delivered properly, was appraised and reported using a step-down approach. The calculation of the overhead costs per day took into consideration the hospital’s actual bed occupancy for the year 2021 by dividing the allocated hospital budget by the bed days per level of care following the guidelines by Drummond et al. [39, 40]. This approach apportioned the actual overhead costs using aggregated cost data for the hospital from the MOH Budget Control and Financial Affairs to obtain the cost per day and multiply it by the recorded length of stay for each patient by their level of care. The components, description, and method of calculating these costs are detailed in [Additional file 3 see Table S7].

### Data analysis

The quantities of each resource input were measured and valued using the specific unit cost from the provider’s perspective. Subsequently, the total costs of each category (consumables, human resources, equipment costs, and overhead) for each patient were summed to obtain the cost for the overall admission for each patient. Descriptive statistics for characteristics are presented as means, standard deviations (SD), medians, and interquartile ranges (IQR) for continuous variables and as numbers and percentages for categorical variables. Given uncertainties in some resource input parameters, a sensitivity analysis was undertaken to assess the robustness of the base case estimates. The sensitivity analysis explored the effect of PPEs, the staff-to-patient ratio, and the use of ventilators on the cost of treating COVID-19 patients. The ± 50% variation threshold for PPE use and staffing levels was selected to reflect plausible fluctuations observed during pandemic surges, informed by procurement records and expert opinion, while ensuring the range remained within realistic operational limits. The cost estimates per admission calculated in the study were then extrapolated to estimate the national-level burden of COVID-19 hospitalisation, by multiplying the total number of admissions in all hospitals in Kuwait by the cost per admission. All cost calculations and figure visualisations were performed using Stata^®^ version 18 (StataCorp LLC, College Station, TX, USA).

## Results

### Patient baseline characteristics

A total of 7569 patients with confirmed COVID-19 were admitted in the hospital and enrolled in the study during the study period, of which 52.8% were female and 47.2% were male (Table 1). The mean age of the patients was 48 years. The age distribution of the patient groups was as follows: 9.1% patients were < 20 years of age, 21.6% were aged between 21 and 40 years, 39.9% were in the 41-to-60-year age category, and 29.3% were aged 60 and above. Most patients were Kuwaiti 78.4%, other nationals representing various nationalities not specifically enumerated constituted the second largest group 10.3%, followed by South Asians 5.9%, and Arabs 5.4%. Thirty- nine% of the patients reported no history of diseases, while 44.8% of patients presented with 1 or 2 comorbidities, and 18.2% had ≥ 3 pre-existing conditions. Additionally, the smoking status “either ever smoked or current smoker” was observed in 3.3% of patients within the total cohort. The uptake of COVID-19 vaccination (1 or 2 doses) among patients before their admission was documented in 22.2% of patients. A notable 22% of patients required ICU admission.

**Table 1:** Patients baseline demographics & hospitalisation characteristics (*N*=7569)

Baseline demographics characteristics		*n* (%)^1^
Age – mean years (SD)		48 (20.5)
Gender	Female	3998 (52.8)
	Male	3574 (47.2)
Age groups, years	1–20	690 (9.1)
	21–40	1642 (21.6)
	41–60	3021 (39.9)
	+ 61	2219 (29.3)
Nationality	Kuwaiti	5935 (78.4)
	Arabs	411 (5.4)
	South Asians	447 (5.9)
	Others	777 (10.3)
Smoking status	No	7325 (96.7)
	Yes	247 (3.3)
COVID-19 vaccinated	No	5887 (77.8)
	Yes	1682 (22.2)
Pre-existing comorbidities	0	2799 (37)
	1–2	3389 (44.8)
	≥ 3	1384 (18.2)
Hospitalisation characteristics		
Length of stay - mean (SD)	General ward	6.8 (6.1)
	ICU	3.3 (9.2)
	Overall	10.1 (10.9)
ICU Admitted, *n* (%)	No	5911 (78)
	Yes	1661 (22)
Discharge status, *n* (%)	Alive	6638 (88)
	Died	933 (12)

Abbreviations: ^1^ The proportion, unless otherwise indicated, *SD* Standard Deviation

The overall mean length of hospital stay was 10.1 days, of which 3.3 days were spent in ICU and 6.8 days were spent in the general ward. 12% of the admitted patients died during hospitalisation, while 88% of patients were discharged alive or transferred to another hospital (Table 1).

### Resource use and hospitalisation costs

**Fig. 1:**
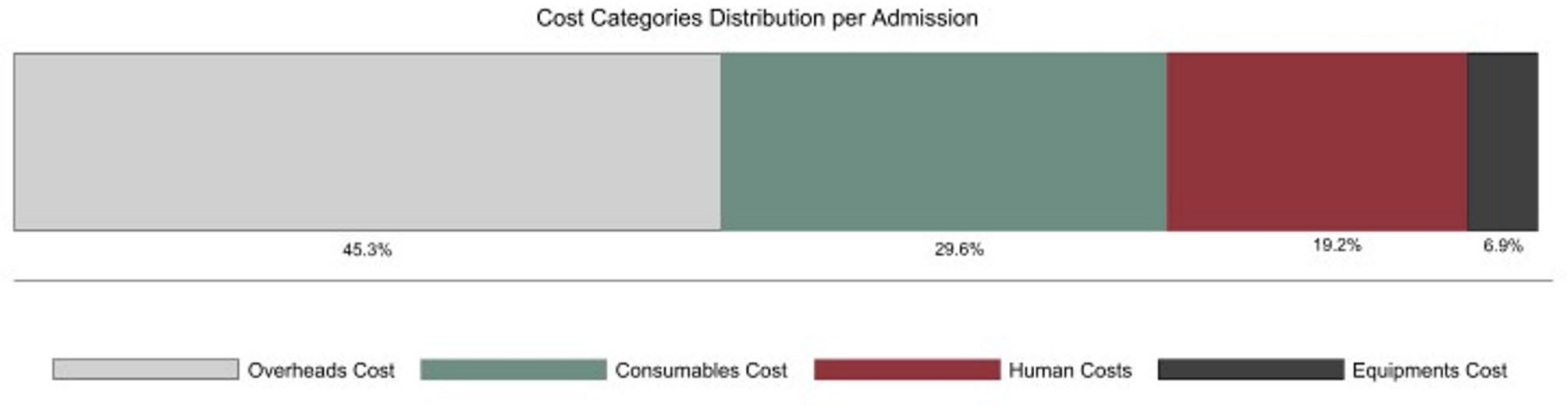
The distribution of cost categories of mean cost per admission

The mean (SD) total costs per admission of COVID-19 patients was $6,599 ($12878), excluding overhead costs (Table 2). When overhead costs were included, the mean (SD) total costs were $12,063 ($18,448). The consumables costs were $3,447 ($7,593) accounting for the highest share 29.6% of the total cost excluding overheads. The second highest category was the human resource costs 19.2%, followed by capital equipment costs 6.9% (Fig. 1). When overhead costs were included, the overhead costs represent the highest share of total costs by 45%.

**Table 2:** Mean Costs & Cost Categories per COVID-19 admission in Kuwaiti Dinars ($PPP)

Costs categories	Mean KD ($PPP)	SD*	Median* (IQR)	%	Category %
Consumables					
Therapeutic agents	415 (2,156)	*(5,558)*	426 (1,478)	62%	
Diagnostics	211 (1,098)	*(1,973)*	469 (725)	32%	
PPEs	38 (193)	*(365)*	74 (101)	6%	
Total Consumables Costs	663 (3,447)	*(7,593)*	1008 (2,488)		29.6%
Human resources					
Physicians	120 (626)	*(1,258)*	208 (286)	27%	
Nurses	245 (1,272)	*(2,382)*	499 (687)	54.8%	
Health professionals	81 (423)	*(689)*	201 (276)	18.2%	
Total Human Costs	447 (2,322)	*(4,318)*	909 (1,238)		19.2%
Capital Equipment					
Medical devices	80 (416)	*(753)*	172 (235)	50.2%	
Ventilators	55 (284)	*(488)*	131 (164)	34.3%	
Beds	25 (129)	*(243)*	50 (68)	15.5%	
Total Capital Costs	160 (829)	*(1,484)*	350 (480)		6.9%
Mean Cost (excl. Overheads)	1,269 (6,599)	*(12,878)*	2286 (4,682)		
Overheads	1,051 (5,465)	*(5,953)*	3741 (4,276)		45.3%
Mean Cost (inc. Overheads)	2,320 (12,063)	*(18,448)*	6306 (8,572)		100%

Abbreviations: *SD* Standard Deviation, *IQR* Interquartile Range, *PPP* Purchasing Power Parity

*Costs in ($PPP)

**Fig. 2:**
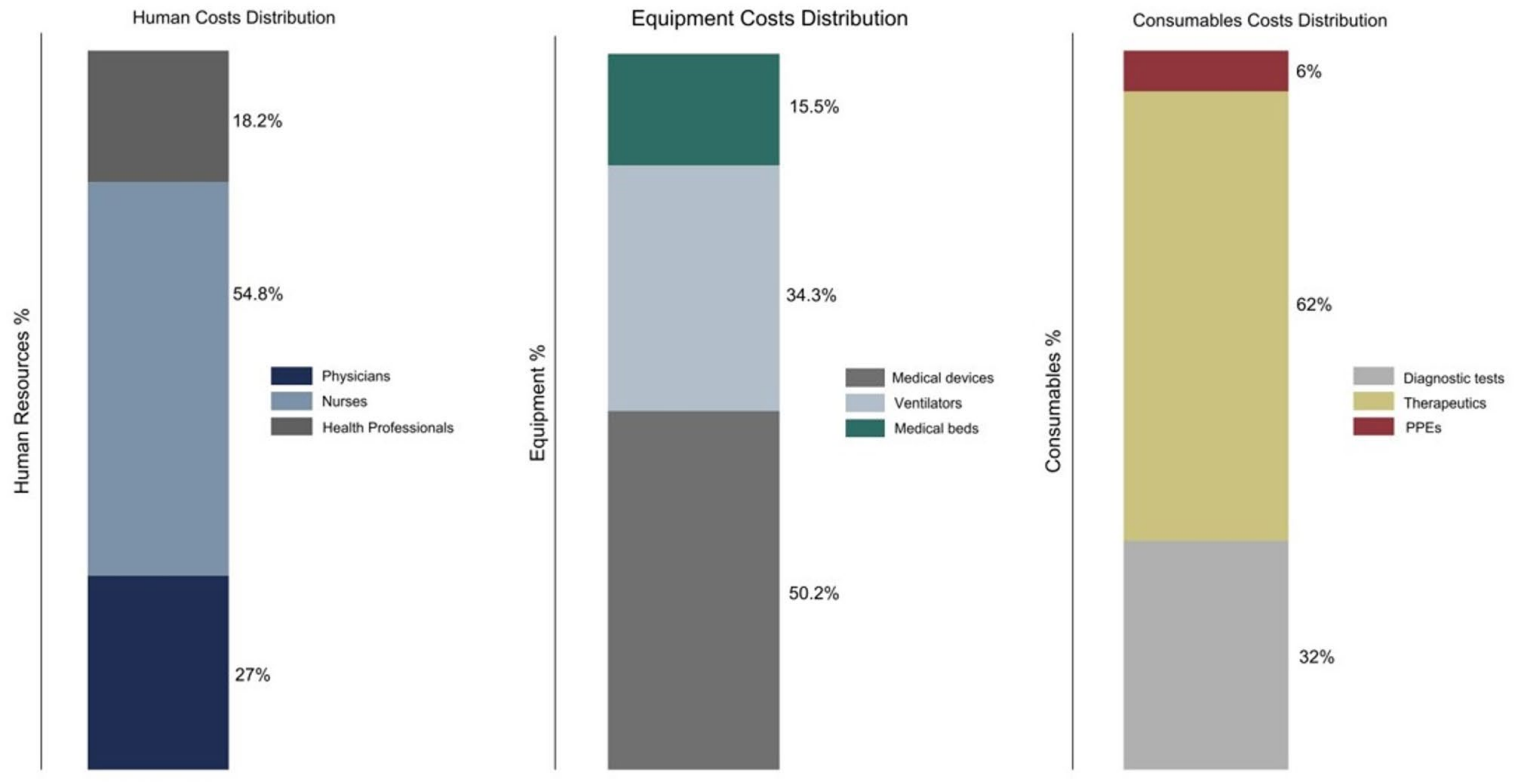
The distribution of cost components among cost categories per admission

Specifically, the breakdown of costs within the consumables showed that the largest cost sub-component within this category was therapeutic agents (e.g. medications) representing 62% of total costs, followed by diagnostics (including laboratory tests and medical imaging) with 32%, and personal protective equipment’s PPEs at 6% (Fig. 2). In the human resources category, nursing costs were the most substantial sub-component, which comprised 55% of the total costs of this category, while physician costs were 27%. Costs associated with allied health professionals, including technicians, lab specialists, pharmacists, and physiotherapists, were 18% of the total human resource cost.

The capital equipment costs per admission was 160 KD (829 $PPP). This category primarily comprises of medical devices, which account for 50% of the total capital equipment costs, costing approximately 80KD (416 $PPP). Cost associated with ventilators, essential for patients requiring respiratory support, constitute 34% of the costs of this category. Costs of medical beds represent the remaining 16% of the capital equipment costs, totalling 25KD (129 $PPP) (Table 2)

### National burden of COVID-19 in Kuwait

The costs estimated from the study was extrapolated to the national level to estimate the national burden of direct costs of COVID-19 in Kuwait. The total number of COVID-19 inpatient discharges in Kuwait was 14,518 in 2021. The national burden of inpatient cases valued using the estimated mean cost per admission from our study (12,063 $PPP) resulted in a total inpatient cost of 189 million $PPP (Table 3).

For context, Kuwait’s population in 2021 was estimated at 4.4 million people. Kuwait’s estimated Gross Domestic Product (GDP) for 2021 was 211,1 billion $PPP, and healthcare expenditure per capita was $2,908 (Table 3).

**Table 3:** Direct health economic burden by Kuwait health indicators in ($PPP)

Kuwait Population, *n* (2021)	4,385,717
Gross Domestic Product	211,1 Bn
Gross Domestic Product per capita	49,669
Health expenditure per capita	2,908
COVID-19 cases, *n*	
Total COVID-19 discharges	14,518
Direct Medical Costs	
Cost per inpatient admission	12,063
Total COVID-19 Direct Medical Costs	189,648,634
Burden (%)	
The ratio of government health care expenditures	2.1%
The ratio of Kuwait’s GDP	0.1 %

Abbreviations: *PPP* Purchasing power parity, *GDP* Gross domestic product, *Bn* Billion

The economic cost of hospitalised COVID-19 patients accounted for 2.1% of the annual government health expenditures. The annual per capita government spending on health in Kuwait was 2,908 $PPP. This indicates that expenditure on hospitalised COVID-19 inpatients was 4.5 times higher than the average per capita health spending. During one year of the pandemic, the direct medical costs of COVID-19 imposed a medical cost burden equivalent to 0.1% of Kuwait’s GDP.

### Sensitivity analysis

**Fig. 3:**
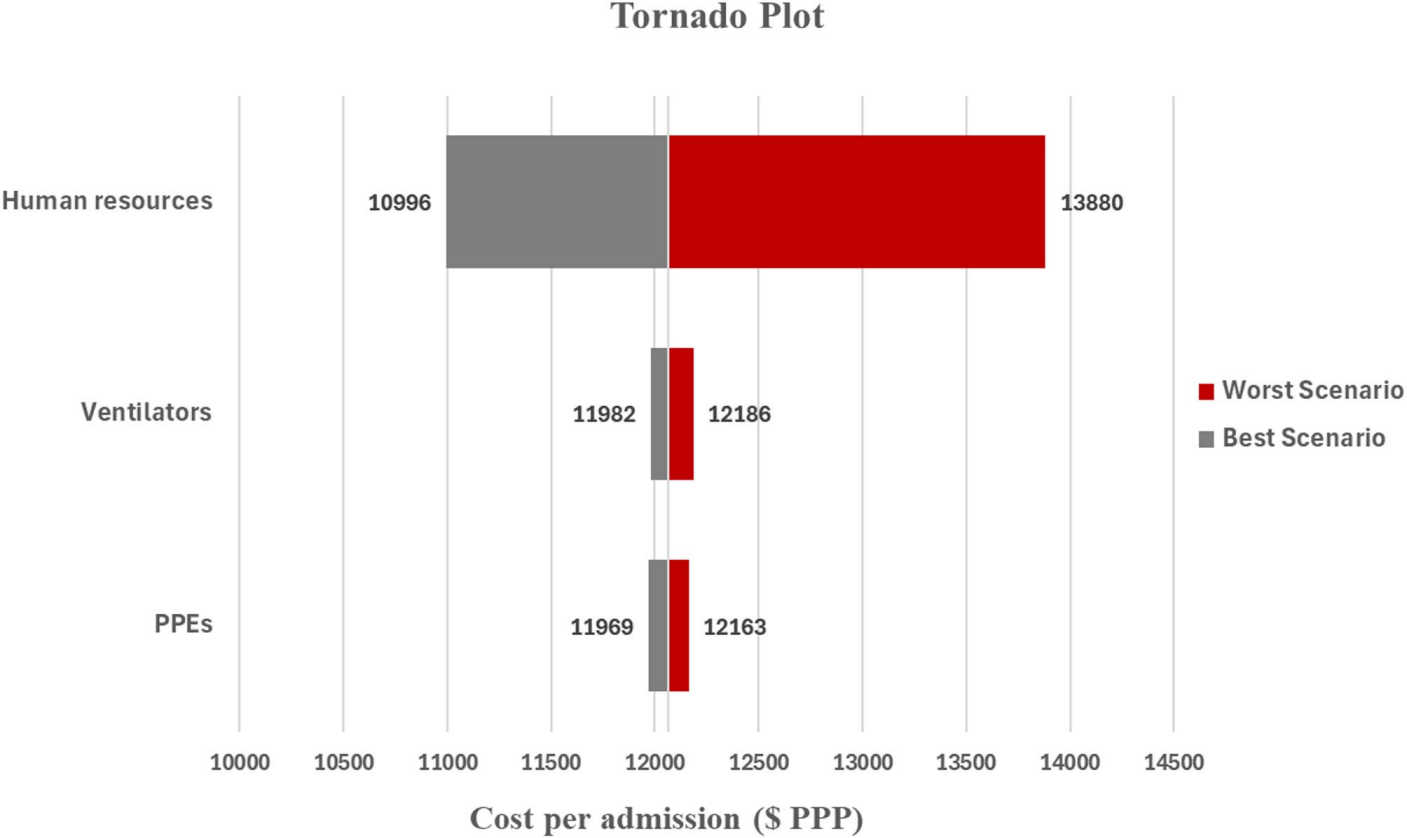
The sensitivity analysis tornado plot

The sensitivity analysis results show that the uncertainty in the human resources detailed in [Additional file 3] had the largest leverage on the mean cost per admission. For example, in worst case scenarios where the staff to patient ratio cost is assumed to be higher by 50% from the base case scenario, the mean cost per admission would increase from 12,063 to 13,880 $PPP. On the contrary, the mean cost per admission would decrease from 12,063 $PPP to 10,996 $PPP when the staff-to-patient ratio was assumed to be 50% lower. Estimates of the uncertainty of the other two cost inputs, the use of ventilators and PPEs, show a slight impact on the mean cost per admission: PPE base cost +50% = $12,163 vs base cost - 50% = $11,969 and ventilators base cost (+50%) = $12,186 vs. base cost (−50%) = $11,982 (see Figure 3).

## Discussion

The study provides a comprehensive analysis of COVID-19 hospitalisations in Kuwait, focusing on patient demographics, resource utilisation, costs, and the overarching national burden of the COVID-19 pandemic associated with hospitalisation. We assessed the direct economic cost encompassing all admitted patients spanning 2021 in a single designated hospital for COVID-19 in Kuwait. The cost of hospitalisation of COVID-19 per patient from the hospital perspective was 2,320 KD (12,063 $PPP). The hospitalisation cost per admission was estimated at 1,266 KD (6,599 $PPP), which increased to 2,320 KD (12,063 $PPP) when overheads are included. This contrasts with another study conducted in Kuwait, which analysed direct costs during the first wave, reporting a higher cost of $16,373 per admission [28]. Our findings also contradict the increase of costs over time in the pandemic. The time-driven increase in the hospitalisation costs was supported by a large study in the US that average hospitalisation costs increased by 26% over a two-year period [41].

The cost structure reveals that overheads were the highest contributor to cost per admission accounting for 45% of costs. In line with this, domination of overhead costs among other cost categories was also found in other studies [42, 43]. This may be attributed to the lower Bed Occupancy Rate (BOR) of the hospital due to the MOH protocol of only admitting COVID-19 patients, resulting in less efficient use of resources. The markedly low occupancy amplified fixed overhead costs per admission, as infrastructure and staffing expenses were spread over fewer admissions. In future planning, flexible bed allocation and adaptable staffing models could help contain such cost inflation during fluctuating demand. The lower BOR could result in higher hospitalisation costs and inversely higher BOR improved efficiency of healthcare facilities resulting beneficial economies of scale [44]. Following overheads, consumables accounted for a considerable part of costs during hospitalisation, which was also observed in other studies [13, 15]. The breakdown of consumable costs indicates that therapeutic agents constitute the most significant proportion as in most studies and are supported by the COVID-19 panel guidelines [13, 15, 34]. This underscores the significant financial burden associated with medications as highlighted elsewhere [13, 45]. These cost estimates could inform future cost-effectiveness assessments of therapeutic strategies in pandemic settings. Given the ongoing shortage of medication within the Ministry of Health, these results serve as a warning: sustainable and continuous supply chain strategies must be a priority in future preparedness efforts. Without proactive measures, healthcare systems may face recurring medication shortages, potentially compromising patient care and escalating costs during future outbreaks. Strengthening supply chain resilience and ensuring the availability of critical therapeutic agents is essential for mitigating financial and operational risks in future public health crises. Furthermore, the significant economic burden of COVID-19 on Kuwait’s healthcare system is apparent, with the annual inpatient costs for the disease at 189 million $PPP. This accounts for 2.1% of the national health expenditure, an estimate that was also in line with findings from Turkey by Oksuz et al. at 2% [32]. In comparison with other high-income countries in the region, Kuwait’s estimated cost per admission is within the range reported for Saudi Arabia (US $11,387–$21,178), reflecting similar healthcare system structures and resource allocation patterns [26]. This aligns with global evidence from a recent meta-analysis, which reported wide variations in direct medical costs, ranging from approximately US $1264 to US $79,315 per patient, with ICU costs up to 3.5 times higher than general ward costs [29]. Another study with findings from China estimated the total societal cost at 2.7% of the GDP [15]. The cost per admission exceeded the government healthcare spending per capita by a substantial margin of 4.5 times higher. However, these estimates don’t include healthcare costs for outpatients, testing, and immunisations. Such findings illustrate the pandemic’s strain on public health resources and stress the urgency for sustainable preparedness strategies.

Our findings reveal critical insights into the characteristics of hospitalised patients, highlighting a predominance of older Kuwaiti individuals, with a significant proportion requiring intensive care. With a substantive cohort of over 7,500 patients, our study underscores the notable impact of COVID-19 on the healthcare system during the 2021 pandemic. The demographic breakdown shows that the majority of patients were between 41 and 60 years of age, suggesting that this group faces a higher risk for severe illness from COVID-19 in line with literature [16, 31]. Moreover, the prevalence of comorbidities in nearly 62% of patients, which was found in other local epidemiological studies earlier in the pandemic [46–48], emphasises the need for continued vigilance and preventive measures for vulnerable populations. Those individuals are typically at greater risk of hospitalisation and adverse outcomes and incur higher inpatient costs [20, 23, 34, 49]. The low vaccination uptake recorded before hospitalisation 22% correlates with higher rates of severe disease, which was also found by Somani et al. [50]. Future research could explore whether vaccinated patients incurred lower hospitalisation costs or experienced reduced severity, which would strengthen the evidence base for vaccination’s economic and clinical benefits. These findings support the uptake of COVID-19 vaccination among patients and reinforce the critical role of vaccination campaigns.

The uncertainty in cost estimates, particularly concerning human resources, underscores the importance of precise staffing and operational protocols during health crises. Adjustments in staff-patient ratios significantly leveraged costs, suggesting that optimising workforce management could lead to substantial savings without compromising patient care quality. This point is particularly relevant for policymakers seeking to understand the economic implications of healthcare staffing decisions during pandemics. A key strength of this analysis is the detailed breakdown of costs per admission into distinct categories. This granularity provides valuable insight into the distribution of healthcare costs, making it possible to identify major cost drivers. Without such breakdowns, cost analyses would be limited to historical references rather than serving as a practical tool for forecasting and planning future resource allocation. Understanding the “behaviour” of unit costs is essential for improving cost-effectiveness and ensuring financial preparedness for future health crises. Such detailed cost data can be linked with clinical outcomes to support future evaluations of the value for money of interventions like ICU care and key therapeutics. While this study provides significant insights, there are limitations worth noting. Reliance on administrative data may leave certain nuances unexamined such us changes in clinical pathways during the hospitalisation, while variations in patient care protocols across different periods could impact the generalisability of the findings. Furthermore, as the analysis was based on data from a single designated COVID-19 hospital, there is a potential selection bias, and the findings may not fully reflect cost structures or patient profiles in other facilities within Kuwait or in different international healthcare settings. However, large data where all hospitalised patients were included in our study, which is a relatively large compared with other studies that relied on a sample of patients or subset of providers [27]. Also, this study utilised real-world data from patient records enhancing the ability to account for case-mix of patients and derive actual use of resources. Our results, which reported all COVID-19 hospitalised patients in 2021, address the limitations present in earlier studies by accounting for changes over time due to pandemic waves, shorter period of analysis, and assessing hospital efficiency in managing patients [13, 27, 28, 37]. This comprehensive approach is particularly crucial given variations in the early waves of the pandemic [28]. Another limitation was the unavailability of respiratory therapy for patients in EHR. Thus, estimates were tested by including ventilators in the sensitivity analysis. The most significant limitation of this study is the exclusion of direct non-medical costs (e.g., transportation, meals, caregiver costs) and indirect costs (e.g., productivity losses, mortality-related and disability-related costs), as well as other costs such as preventive measures, outpatient care, long-COVID management, and non-pharmaceutical interventions, which were not included and were beyond the scope of this research. Excluding these categories likely leads to an underestimation of the total economic burden of COVID-19, as societal losses extend beyond hospital-based medical expenses. Future research could delve deeper into the sub-group variations in terms of costs, their impact on treatment efficacy and resource allocation, and the long-term health outcomes for individuals’ post-infection.

## Conclusion

The insights derived from this study emphasise the critical need for targeted public health interventions, resource allocation strategies, and economic planning. By identifying and addressing the primary cost drivers associated with COVID-19 hospitalisations, Kuwait’s healthcare authorities can enhance preparedness for future healthcare challenges while ensuring sustainability in health spending.

## Supplementary Information

Supplementary Material 1

## Data Availability

The availability of these data is not publicly available. However, data are available from the corresponding author upon reasonable request and with permission from the MOH Research and Ethics Committee.

## Abbreviations

MOH: Ministry of Health
EHR: Electronic Health Record
ICU: Intensive Care Unit
PPE: Personal Protective Equipment
GDP: Gross Domestic Product
KD: Kuwaiti Dinars
PPP: Purchasing Power Parity
SD: Standard Deviation
BOR: Bed Occupancy Rate
IQR: Interquartile Range
