# Supplementary material for: Estimating the direct medical cost of illness of COVID-19 hospitalisations in Kuwait: efficiency trade-offs from real-world data analysis
Source: Health Econ Rev. 2025 Nov 19;15:99. doi: 10.1186/s13561-025-00694-9 (PMC12628864; doi:10.1186/s13561-025-00694-9)
Supplement: Supplementary file 1 — Supplementary Material 1 [file 13561_2025_694_MOESM1_ESM.docx]

# Additional File

This additional material details the methodology applied to estimate resource use and costs associated with COVID-19 inpatient care. It describes the data sources, unit cost derivation, and calculation approaches for diagnostics, therapeutics, personal protective equipment (PPE), and capital equipment across levels of care (general ward and ICU). All assumptions, costing parameters, and occupancy adjustments are specified to support the main analysis.

# Diagnostics

This section provides a detailed description of the inputs used to estimate the costs of consumables per patient per day for each level of care – general ward and intensive care units (ICU). It refers to all diagnostics performed and utilized to treat the hospitalized patients. It includes laboratory tests conducted from the following laboratory sections: microbiology, haematology, virology, and biochemistry. The cost of tests was obtained from the General Medical Stores Department in (MOH). The radiology tests, consumables used, including contrasts, plastic covers of the cassettes/detectors, equipment capital cost, and human resource time costs, were measured to obtain the unit cost per test. The cost of diagnostic tests was calculated by counting the quantity (q) of each test ordered for the patient multiplied by the respective unit cost (p) as illustrated by Vassall et al. (1). The unit costs used for the analysis for diagnostics were obtained from the Kuwait (MOH) Budget Control and Financial Affairs sector are shown in (Table S1).

Table S1. Inputs for Laboratory Diagnostic Costs in Kuwait Dinar (int$ PPP 2021)

| (1)  Resource input | (2)  Unit cost  in K.D.(PPP$) | (1)  Resource input | (2)  Unit cost  in K.D.(PPP$) |
| --- | --- | --- | --- |
| PCR Sars-Cov2 | 10 (51) | Troponin | 4 (21) |
| Complete blood count | 4 (21) | MRSA culture | 4 (21) |
| ESR | 3 (16) | MRSA Geneexpert | 5 (26) |
| ABG | 3 (16) | MDRO | 6 (31) |
| Blood culture & sensitivity | 9 (47) | Lactic acid | 3 (16) |
| Bone profile | 6 (31) | Sputum culture | 5 (26) |
| CRP | 5 (26) | LDH | 6 (26) |
| Coagulation profile | 9 (46) | Ferritin | 3 (16) |
| D-Dimer Assay | 4 (21) | Thyroid profile | 15 (78) |
| Liver function (LFT) | 9 (47) | PCT | 3 (16) |
| Ferritin | 3 (16) | Liver function (LFT) | 9 (47) |

^1^ The dosage and frequency of resource utilization were calculated on a case-by-case basis according to (EHRs).

^2^ Kuwait (MOH) Budget Control and Financial Affairs

### Therapeutics

The detailed medications prescribed for each patient during the episode of hospitalization were extracted from (EMR). The form of medications given to patients was either oral or/and parenteral. Several quantities and volume levels of therapeutics were administered on a case-by-case basis. The medications’ costs were summed up for each patient for the entire episode of treatment by factoring the volume of dosage, the frequency per day, and the duration of the treatment regimen for each treatment input. Patients with comorbidities and those who were critically ill were merited extensive use of medications due to complications from COVID-19 illness. The total number of medications captured in our analytical dataset for the parenteral and oral medications were 193 and 247, respectively. The most frequent medications used in the management of COVID-19 patients were categorized into four major groups: antibiotics/antimicrobials, anticoagulants, corticosteroids, and supportive/nutritional agents. The unit costs of the medications used were obtained from the medications procurement list in the General Medical Stores Department (MOH) and are displayed in (Table S2).

Table S2. Inputs for Therapeutic Costs in Kuwait Dinar (int$ PPP 2021)

| Resource input ^a^ | medication | Dose ^a^ | Unit cost ^b^  in K.D.(PPP$) |
| --- | --- | --- | --- |
| Antibiotics/Antimicrobials | Ceftriaxone | 2g IV / day | 0.5 (2.6) |
|  | Moxifloxacin | 400 mg | 4.4 (22.8) |
|  | Levofloxacin | 750mg IV / day | 1.3 (7.1) |
|  | Tocilizumab | 400 mg / one dose | 241 (1248) |
| Corticosteroids | Dexamethasone (prednisone 40 mg) | 6mg Daily IV  or 10 days for patients on ventilation /ECMO | 0.8 (4.1) |
| Anticoagulation | Enoxaparin | Regular dose: 40mg | 1.2 (6.2) |
|  |  | High dose: 120mg | 2 (10.3) |
| Antiviral | Remdesivir | Adult (wt > 40 kg): 200 mg IV loading dose on day 1, then 100 mg IV daily maintenance dose. | 125 (647.5) |
| Eternal nutrition | Normal Saline 500ml | * | 0.2 (1) |
|  | Dextrose 500ml |  | 0.2 (1) |
| Supportive Care | Vitamin D |  | 0.3 (1.5) |
|  | Zinc Lonzenges |  | 0.1 (0.5) |
|  | Vitamin C |  | 0.02 (0.1) |

^a^ Electronic medical records (EHRs)

^b^ Kuwait (MOH) General Medical Stores Department

* The dosage and frequency of resource utilization were calculated on a case-by-case basis according to (EHRs).

### **Personal Protective Equipment (PPEs)**

The quantities of personal protective equipment (PPEs) needed for staff were obtained through staff-patient ratio, while guidelines for the types to be used were obtained from the Kuwait (MOH) COVID-19 Management Protocol which we will refer in this section as “Protocol”. The protocol encouraged adherence in the use of medical masks by health and clinical workers in all clinical settings throughout routine round-activities. Only the (PPEs) used by clinicians who were in direct contact with COVID-19 patients were considered in this section. The (PPE) types that were required to be worn in wards where aerosol-generating procedures (AGPs) were performed were considered in-line with recommended infection prevention control practice guidelines by the protocol.

The basic (PPEs) attire that should be worn for each staff was classified according to level of care (general ward or ICU), whereas the (PPEs) needed to conduct a specific procedure were mentioned and quantified in the previous section. Medical masks, non-sterile gloves, and gowns were the basic staff gear for general wards. The ICU personnel follow the same basic attire in addition to sterile gloves, gowns, cap, show covers, and (N95) masks which were scarce and limited to the ICU wards where aerosol generating procedures were performed.

The quantities of (PPEs) needed for patient per day were calculated based on the assumption that the clinician would use the (PPE) for the entire day (except the gloves) which were required to be replaced in between treating patients. This assumption was applied in the general ward and ICUs with a capacity of 25 and 10 patients, respectively, and in accordance with staff to patient ratio. The quantities of the daily (PPEs), considering the respective costs per patient day are shown in (Table S3).

Table S3. Costs for Consumables per staff per day in Kuwait Dinar (int$ PPP 2021)

| (1)  Resource input | (2)  Unit cost | Level of Care | Quantity per patient day ^c^ | Cost per patient day in K.D.(PPP$) |
| --- | --- | --- | --- | --- |
| Non-sterile gloves | 0.02 | GW | 24 (15-36) | 0.5 (2.5) |
|  |  | ICU | 48 (36-72) | 1 (5) |
| Sterile gown | 0.6 | GW | 2 (1-3) | 1.2 (6) |
|  |  | ICU | 4 (3-6) | 4 (12.5) |
| Face masks | 0.01 | GW | 2 (1-3) | 0.02 (0.1) |
|  |  | ICU | 3 (2-4) | 0.03 (0.2) |
| Sterile gloves | 0.02 | ICU | 48 (36-72) | 1 (5) |
| N95 | 1 |  | 3 (2-4) | 3 (15.5) |
| Cap | 0.025 |  | 4 (2-4) | 0.1 (0.4) |
| Show cover | 0.01 |  | 5 (2-4) | 0.03 (0.6) |

^1^ Kuwait (MOH) COVID-19 Management Protocol

^2^ Kuwait (MOH) Budget Control and Financial Affairs

^c^ Assumption based on the number of the staff to patient ratio

# Capital Equipment

This section provides a detailed description of the inputs used to estimate the costs of capital equipment per patient per day for each level of care – general ward and intensive care units (ICU). A description of capital equipment that has a useful life and can be utilized for more than one year is presented in (Table 8) as the cost per day in Kuwaiti Dinars and (int$ PPP 2021). The resources used during the episode of care were identified and the quantities of resources were grouped based on the level of care. All equipment were set to have a useful life of ten years based on the biomedical engineering department guidelines in the MOH. Thus, all equipment costs were annualised by applying the annualiseation factor that cooresponds to ten years useful life. The unit cost of the item was divided by 7.72% with a discount rate of 5% to estimate the cost per year (2). To estimate the economic cost per day for each device, the annualised cost was divided by the actual occupancy-driven days for each level of care as detailed in (Table.7). This approach provides economic cost inputs and clearly depicts the opportunity cost forgone by allocating hospital services solely to treating COVID-19, following the guidelines in Creese et al. (3).

Occupancy rates of the hospital during the year 2021 were obtained from the hospital medical records statistics. The patient bed days were (26496), (60042 ) and (86538 ) bed days for the ICU, general wards, and in total, respectively. The total bed capactiy of the hosital is (1117), (196) of which occupy the ICUs and (921) of which are used in the general ward. By multiplying each level beds with (365) days, then total hospital capacity in its full efficiency for each level is obtained. [1117 beds x 365 days = 407705 bed days], [196 x 365=71540 bed days], and [921 x 365 = 336165 bed days].

Table S4. The Hospital Occupancy Rates

| Total capacity | Beds **A** | Hospital occupancy  **(A x 365) B** | Total patient bed days **C** | Occupancy rate **(C/B)** |
| --- | --- | --- | --- | --- |
| General wards | 921 | 336165 | **60042** | 18% |
| ICUs | 196 | 71540 | **26496** | 37% |
| Total | 1117 | 407705 | **86538** | 21% |

The general ward occupancy for the year 2021 was (60042 bed days) divided by total number of the hospital yearly occupancy for the general wards (336165). This estimation showed that the actual occupancy rate was 18%. Thus, by multipying this rate with (365) days a year (365 x 18% = 66 days) will yield (66) days. Therfore, the economic cost per day for the general ward equipment would be the annualised unit cost of the equipment divided by (66) days. Whereas, the economic cost for the ICU devices would be the annualised cost divided by (135) days, the actual occupancy-driven days (365 x 37%=135 days). The hospital financial cost was also reported by applying the straightforward depreciation method of dividing the equipment unit cost by ten years then by 365 days. The estimated financial cost was multiplied by the quantity of the equipment used per one patient per day. Both costs are reported in (Table S5).

Table S5. Inputs for Capital Equipment Costs per day in Kuwait Dinar (int$ PPP 2021)

| Resource input | Ward | (1)  Quantity | (2)  Unit Cost (Average) | Annualised Cost | Economic Cost per day (PPP $) | Financial cost (int$) | |
| --- | --- | --- | --- | --- | --- | --- | --- |
| Syringe pump | GW | 1 | 505 | 65 (339) | 1 (5) | 0.1 (0.7) | |
|  | ICU | 4 |  |  | 1.9 (9.8) | 0.6 (3.1) | |
| Infusion pump | All | 1 | 745 | 96 (500) | 1.2 (6.5) | 0.2 (1) | |
| BiPAP/CPAP ventilator | ICU | * | 6487.5 | 840 (4353) | 6.2 (32) | 1.8 (9.2) | |
| High flow machine | GW | 1 | 2302.5 | 298 (1545) | 4.5 (23.5) | 0.6 (3.2) | |
| Feeding pump | ICU | 1 | 145 | 19 (98) | 0.1 (0.7) | 0.04 (0.2) | |
| ECMO machine | ICU | 1 | 69250 | 8970 (46466) | 66.5 (344) | 19 (98.2) | |
| Portable monitor | All | 0.04 ^a^ | 3930 | 509 (2637) | 0.3 (1.4) | 0.04 (0.2) | |
| Patient monitor | GW | 0.16 ^b^ | 4617.5 | 598 (3098) | 1.5 (7.5) | 0.2 (1) | |
|  | ICU | 2 |  |  | 8.8 (45.5) | 2.5 (13) | |
| Mechanical Vent | ICU | 1 | 14900 | 1930 (9998) | 14.3 (74) | 4.1 (21.1) | |
| Electric bed | GW | 1 | 627 | 81 (421) | 1.2 (6.5) | 0.2 (0.9) | |
|  | ICU | 1 | 5200 | 673 (3489) | 5 (26) | 1.4 (7.4) | |
| Suction pump | ICU | 1 | 1403 | 182 (941) | 1.3 (7) | 0.5 (2) | |
| Laryngoscope | ICU | 0.1 ^c^ | 10696.5 | 1385 (7177) | 1 (5) | 0.3 (1.5) | |
| ^1^ Expert opinion.  ^2^ Kuwait (MOH) General Medical Stores Department.  * The quantity of utility is based on the resource use per patient in the medical record. | | | | | | |  |
| ^a^ 2 monitors used in case the patient has to be moved. | | | | | | |  |
| ^b^ 4 monitors for each ward shared amongst 25 patients. | | | | | | |  |
| ^c^ One is required for each ICU ward, and divided by 10 the number of ICU beds, and needed only one time. | | | | | | |  |

# Human resources

The human resources cost inputs were derived from the time spent for each cadre involved in the medical care. The annual salaries were obtained from the sources mentioned above and the breakdown of daily and hourly wage was calculated. The total working hours per week were 40 hours, and the cost per hour for both remuneration schemes (national/non-national staff) were presented. The nationals/non-nationals ratio (1/3) was the reference to estimate weighted cost per hour as the base case scenario (Table S6). The upper and lower bounds of cost per hour were used in the sensitivity analysis.

The staff to patient ratio was calculated by dividing the number of patients in each ward (general ward 25, and ICU 10) by the actual number of staff in each ward according to each cadre. In detail, there were 6 nurses in each general ward taking care of the 25 patients which yield ( 25/6 = 4.1) as 1 nurse for every four patients. For the ICU, the critical nature of conditions necitate the availability of one nurse for every ICU patient. Medical doctors were distributed according to the number and the wards they were able to cover. Each consultant covered 4 general wards with one round in the beginning of the shift and one at the end of the day. At the same time, each consultant was required to manage and cover one ICU ward during their duty.

## Overheads

the cost of overheads, which is the cost of preparing a setting in which health services can be delivered properly was appraised and reported using step-down (top-down) approach. This approach apportioned the actual overhead costs using aggregated cost data for the hospital from MOH Budget Control and Financial Affairs to obtain the cost per day and multiply it by the length of stay for each patient by level of care. These costs included the annual salaries, operational non-salary expenses, and the general non-medical equipment expenditure for the year of 2021. The overhead costs were for the following sections: administration, maintenance, security, information technology, general non-medical stores, infection control, dietary/kitchen, preventive medicine, biomedical engineering, center sterilization unit, and hygiene/cleaning services.

The ratio of the total number of patient days during 2021 for general wards and ICU stays was used as an apportionment factor for the overhead costs per day of stay. The total number of patient days was 86539 days, with a 2 to 1 occupancy ratio (GW/ICU). Apparently, the segregated patient days were 26496 (31%) and 60042 (69%) for the ICU and GW, respectively. When applying the same ratio as an apportionment factor on the total expenditure of the overheads, a total of 8,953,599 million KWD, the cost per day for ICU would yield 2,775,616 KWD / 26496 patient days = 105 KWD (544$PPP) , and the cost per day for the general ward would be 6,177,983 KWD / 60042 patient days = 103 KWD (534$PPP).

| Medical staff | (1)  Level of care | (1)  Staff/Patient ratio | (2)  Hourly wage (Weighted) | Base Case Cost per day (int$ PPP) | Best Case  Cost per day (int$ PPP) | Worst Case  Cost per day (int$ PPP) |
| --- | --- | --- | --- | --- | --- | --- |
| Registrar (internal medicine) | GW | 1 : 25 | 12 (64) | 4 (21) | 3 (13) | 5 (26) |
|  | ICU | 1 : 10 |  | 10 (52) | 6 (33) | 13 (67) |
| Consultant (internal medicine) ^a^ | GW | 1 : 100 |  | 1 (5) | 1 (3) | 1 (5) |
|  | ICU | 1 : 10 |  | 10 (52) | 6 (33) | 13 (67) |
| Intensivist ^c^ (ICU) | ICU | 1 : 30 |  | 3 (16) | 2 (11) | 4 (21) |
| Cardiovascular specialist a | ICU | 1 : 30 |  | 3 (16) | 2 (11) | 4 (21) |
| ICU nurses ^a^ | ICU | 1 : 1 | 6 (32) | 49 (254) | 32 (166) | 75 (389) |
| Ward nurse ^b^ | GW | 1 : 4 |  | 12 (62) | 8 (41) | 19 (98) |
| Physiotherapist | GW | 1 : 100 |  | 0.5 (3) | 0.5 (2) | 1 (5) |
|  | ICU | 1 : 20 |  | 2.5 (13) | 2 (8) | 4 (21) |
| Respiratory specialist | GW | 1 : 25 |  | 2 (10) | 1 (7) | 3 (16) |
|  | ICU | 1 : 10 |  | 5 (26) | 3 (17) | 8 (41) |
| Pharmacist | All | 1 : 80 |  | 0.6 (3) | 0.5 (2) | 1 (5) |
| Laboratory specialist |  | 1 : 40 |  | 1 (5) | 1 (4) | 2 (10) |

| ^1^ Expert opinion  ^2^ Kuwait (MOH) Budget Control and Financial Affairs  ^a^ Consultants were leading the medical team in ICU, and a shared role with intensivist, internal medicine specialist, and CV specialist. |
| --- |
| ^b^ One nurse is assigned with each ICU patient |
| ^c^ six nurses were assigned to a ward with 25 patients |
| ^*^ All calculations were based on working 8 hours/day full time. |

Table S6. Inputs for Human Costs in Kuwait Dinar per patient day (int$ PPP 2021)

Table S7. Cost inputs for overheads per day in Kuwait Dinar per patient day (int$ PPP 2021)

| Level of care | Total patient days (A) | Apportionment  (B) | Total overhead costs **(8,953,599 KD**) **x B** (C) | Cost per bed day **(C/A)** |
| --- | --- | --- | --- | --- |
| General wards | 60042 | 69% | KWD 6,177,983 | 103 (534) |
| ICUs | 26496 | 31% | KWD 2,775,616 | 105 (544) |
| Total | 86538 | 100% | 8,953,599 KD |  |

# References

1. Vassall A SS, Kahn J, et al. Reference Case for Estimating the Costs of Global Health Services and Interventions. Global Health Cost Consortium; 2020.

2. Drummond MF, Sculpher MJ, Claxton K, Stoddart GL, Torrance GW. Methods for the Economic Evaluation of Health Care Programmes: Oxford University Press; 2015.

3. Creese AL, Parker D, World Health O. Cost analysis in primary health care : a training manual for programme managers / edited by Andrew Creese and David Parker. Geneva: World Health Organization; 1994.
